# The Efficacy of Dog Assisted Therapy in Detained Drug Users: A Pilot Study in an Italian Attenuated Custody Institute

**DOI:** 10.3390/ijerph14070683

**Published:** 2017-06-24

**Authors:** Laura Contalbrigo, Marta De Santis, Marica Toson, Maria Montanaro, Luca Farina, Aldo Costa, Felice Alfonso Nava

**Affiliations:** 1 Italian National Reference Centre for Animal Assisted Interventions, Istituto Zooprofilattico Sperimentale delle Venezie, Viale Dell’Universita 10, 35020 Legnaro (Padua), Italy; mdesantis@izsvenezie.it (M.D.S.); mtoson@izsvenezie.it (M.T.); maria.montanaro@libero.it (M.M.); lfarina@izsvenezie.it (L.F.); 2 Veterinary Service, Local Health Unit n. 6-Euganea, Via Fra’ Paolo Sarpi 76, 35100 Padua, Italy; aldo.costa@aulss6.veneto.it; 3 Prison Healthcare Service, Local Health Unit n. 6-Euganea, Via Temanza 2, 35100 Padua, Italy; felicealfonso.nava@aulss6.veneto.it

**Keywords:** drug addiction, inmates, dog assisted therapy, animal assisted interventions

## Abstract

Drug addiction is a major care and safety challenge in prison context. Nowadays, rehabilitation and specific therapeutic programs are suggested to improve health and well-being of inmates during their detention time and to reduce substance abuse relapse after release from prison. Among these programs, several studies reported the benefits for inmates coming from animal assisted interventions. In this pilot controlled study, we investigated the efficacy of a dog assisted therapy program addressed to 22 drug addicted male inmates housed in an attenuated custody institute in Italy. The study lasted six months, the treated group (12 inmates) was involved once a week for one hour in 20 dog assisted therapy sessions, whereas the control group (10 inmates) followed the standard rehabilitation program. One week before the beginning and one week after the end of the sessions, all inmates involved were submitted to symptom checklist-90-revised and Kennedy axis V. Inmates involved in the dog assisted therapy sessions significantly improved their social skills, reducing craving, anxiety and depression symptoms compared to the control group. Despite the limitation due to the small number of inmates enrolled and to the absence of follow up, we found these results encouraging to the use of dog assisted therapy as co-therapy in drug addicted inmates rehabilitation programs, and we claim the need of more extensive study on this subject.

## 1. Introduction

### 1.1. Drug Addiction as Health Challenge in Prison Settings

Drug addiction represents a major health care and safety challenge in U.S., Canadian and European prisons. A 2004 survey by the U.S. Department of Justice estimated that about 70% of State and 64% of Federal inmates regularly used drugs before incarceration [1]. A recent Canadian report found that the prevalence rate of substance use disorders among prisoners is 49.6% [2]. These data are confirmed by European statistics: although most prisoners stop their drug use while incarcerated, studies in 15 European Union (EU) countries show that between 2% and 56% of prisoners reported drug consumption even during detention. In addition, among those imprisoned for drug-related crimes and not treated during their prison term, the majority relapse within the year following release [3]. A recent study confirms that 60% of former inmates met at least one criterion of the Diagnostic and Statistical Manual of Mental Disorders (DSM 5) for substance dependence at one year post-release [4].

Drug addiction arises with physiological, behavioural and cognitive symptoms that find their origin in genetic, biological, psychological and social causes [5]. It starts as a rewarding behaviour (involving the ventral striatum) and evolves to a compulsive behaviour (implicating the dorsal striatum) associated to Pavlovian-conditioned stimuli showing onwards psychological deficits, including poor social skills and emotion regulation, alexithymia, prevalent impulsive action and impulsive choice [6,7,8]. Symptoms arise because of drug-induced pharmacological changes of specific brain circuits (cortico-basal-ganglia-thalamo-cortical, limbic-basal-ganglia and limbic-cortical), which are implicated in associative learning, reward mechanisms, goal directed behaviour, behavioural habit formation, stress, cognition and emotion control [9,10]. During detention, these psychological deficits can even worsen in prisoners, facilitating their relapse in the drug abuse after release or increasing the use of substances during detention when available [11,12,13].

### 1.2. Animal Assisted Program in Prison Settings

In the prison context, inmates affected by drug addiction need to comply with detention life, characterized by heavy emotional entailments and by the customs and culture of the correctional facility they must take on. Therefore, in order to adapt to this difficult environment, full of stressors and infightings, they have to develop their own coping strategy, which often results in being dysfunctional because of their addiction [14,15]. In this framework, rehabilitation and specific therapeutic programs are strongly recommended to facilitate the reintegration of inmates into society as law abiding citizens, overcoming the concept of prison term as a punishment and enhancing a health-promoting approach to offenders’ management in prison settings [16]. Time spent in prison offers an opportunity to influence the future lives of inmates, making a major contribution to improving health and well-being of these disadvantaged people, reducing substance abuse relapse as well as financial cost for the community and preventing future criminal behaviour [17,18,19]. In particular, animals appear to be increasingly incorporated into correctional programs in prison as part of vocational and social skills training [20] and in the treatment of substance dependence [21,22]. Several studies have reported positive psychological and physiological effects of prison-based animal programs and animal assisted interventions with inmates, which confirm the general idea that human–animal interactions contribute to human health and well-being [23,24,25,26,27,28]. However, even if they have shown promising results, only Japerson (2010) and Burger (2011) went through health outcomes of animal assisted therapy with drug addicted inmates [29,30]. In light of the examined literature, the primary aim of our controlled trial was to investigate the effect and the efficacy of a dog assisted therapy (D.A.T.) program addressed to a cohort of drug addicted male inmates housed in the attenuated custody institute of the Italian prison “Due Palazzi” in Padua (North-Eastern Italy). We analyzed the role of D.A.T. as a co-therapy to improve inmates’ level of functioning and to reduce their psychological distress. The primary endpoint with respect to efficacy was a statistical significant difference in psychological functioning improvement and distress symptoms’ reduction in inmates involved in D.A.T. compared to those not exposed.

## 2. Materials and Methods

### 2.1. Ethical Statements

This study was conducted in accordance with the Declaration of Helsinki and the Italian Law on privacy of personal data. All subjects involved gave their written informed consent for the inclusion in the study. The protocol was approved by the Scientific and Ethics Committee of the Istituto Zooprofilattico Sperimentale delle Venezie (Project Identification Code: 8/14).

### 2.2. Study Design

We used an unmatched controlled study design (new intervention + treatment-as-usual vs. treatment-as-usual) that is appropriate to investigate the effects of a specific health intervention like D.A.T. in a population, in our case a cohort of drug addicted male inmates. This study design needs to answer a practical question of whether D.A.T., which was added to the standard rehabilitation program of the attenuated custody institute thanks to a limited regional funding, provided more beneficial results compared to the standard program. This approach ensured benefits to all participants of the study without the risk of patients receiving a new treatment of unknown utility [31]. The D.A.T. program lasted six months (from October 2015 to March 2016) and data were collected one week before the first D.A.T. session and one week after the end of the program.

### 2.3. Setting

The attenuated custody institute of the Italian prison “Due Palazzi” in Padua (North-Eastern Italy) houses 35 male inmates affected by drug addiction. It provides programs to rehabilitate them towards psychological, educational and medical support, in a secure environment. The health care taker staff includes medical doctors, psychotherapists and psychologists working in partnership with educators to assure that all social and health problems of inmates are faced properly. The institute has its own rehabilitation program that aims to manage inmates effectively, reducing the risk of re-offending behaviour and facilitating their reintegration in the society. The program includes educational and working activities as well as psychodiagnostic and therapeutic ones (e.g., relaxation techniques, stress and anxiety management course, recognition and management of emotions training, and assertive training).

The program takes place in prearranged phases:

1. selection of inmates with the suitable features to be involved in the program by the prison health care unit and the prison administration;
2. subscription of the “therapeutic-rehabilitative agreement” by the inmate;
3. observation and psycho-diagnostic evaluation of the inmate by the clinical and functional point of view, taking into consideration his personal resources;
4. planning of the “individual therapeutic program” (I.T.P.);
5. integration of the patient in care and rehabilitative activities;
6. evaluation of results reached by the patient compared to the fixed aims.

The standard program is comprehensive of the following activities:

✓ individual interviews with a psychotherapist once a week (1 h or more according to patients’ needs);
✓ psychotherapy sessions in small group focused on emotion control and assertiveness (1 h, twice a week);
✓ relaxation techniques and stress management course (2 h, once a week);
✓ educational activities: design and painting course, theatre course (3–4 h, once a week);
✓ sport activities (rugby), (2–3 h, twice a week).

Recently, D.A.T. sessions have been added to the standard rehabilitation program as an additional co-therapy to which inmates can choose to take part.

### 2.4. Dog Assisted Therapy Sessions

The D.A.T. sessions we considered in this study were performed following the Italian National Guidelines for animal assisted interventions [32], which establish the presence of at least two figures on the setting to manage an animal assisted therapy session: the healthcare professional (in our case a psychotherapist) and the animal handler. We enrolled one psychotherapist with specific training and 10-year expertise on dog assisted interventions to manage all D.A.T. sessions. The dog-handler couples were selected through simulation tests. Eventually, handlers should pass an interview with a psychologist and the medical doctor responsible for the project.

Dogs involved in the D.A.T. program were selected after a clinical and behavioural examination performed by a veterinarian with expertise in animal assisted interventions. They were all adult animals, belonging to medium-big size breeds, in good health condition, well socialized and specifically trained to perform dog assisted interventions with various kinds of patients. Health and welfare conditions of dogs were monitored by the veterinarian of the team (in collaboration with dog handlers) during the whole project.

Thanks to periodic team meetings, before and during the study, dog handlers received instructions from the psychotherapist and the medical doctor on activities to perform with dogs during the sessions and feedback about their performances.

**Figure 1:**
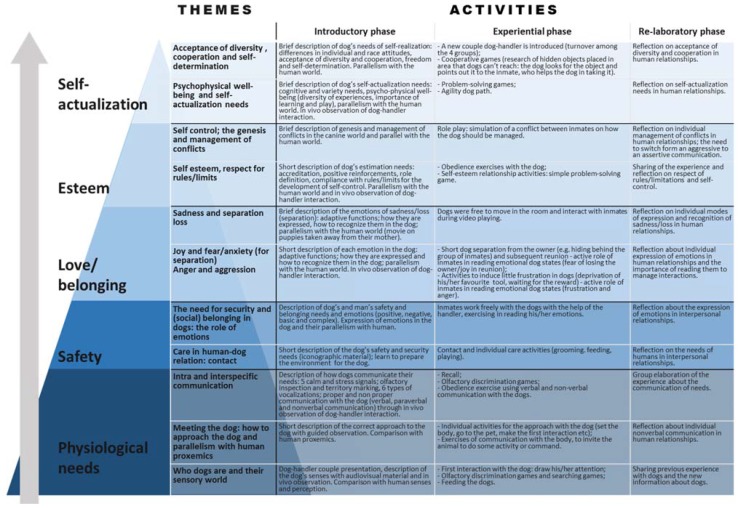
Activities performed during dog assisted therapy (D.A.T.) sessions.

Treated group was divided in four small sub-groups of three people and each sub-group was randomly assigned to a dog-handler couple. Each group was involved in a 60’ session of D.A.T., once a week for six months. Every session followed a standard scheme: an introduction phase during which the psychotherapist introduced the theme and patients were engaged in observational activities; afterwards, a phase in which they actively experienced the interaction with the dog and they were involved in management and performance activities; finally, the elaboration of the experience with the support of the psychotherapist. The psychotherapist previously set all of the activities performed with the dog handlers. They were analogous for all the sub-groups and they were structured following the Maslow’s hierarchy of needs, also known as the Maslow’s Pyramid [33]. At the beginning, the sessions were focused on the base of the pyramid (physiological needs), while going on with the sessions, they moved towards the apex of the pyramid up to self-realization, exploiting the parallelism between dog and human needs and behaviour. During all sessions, dogs were free to interact with people and they were kept on leash only when the activities required it. All activities performed during D.A.T. sessions are described in Figure 1.

### 2.5. Study Population and Eligibility Criteria

Our study population was an open cohort composed of 20–40 year old male inmates, coming from Italy, North Africa or Eastern Europe. They were all naturalized Italian citizens living in Veneto region sentenced to short prison terms (not less than one year and not more than four years) to spend in the attenuated custody institute of the Italian prison “Due Palazzi” in Padua (North-Eastern Italy). All of them were diagnosed for substance-related and addictive disorders as defined by DSM-5. They had no other mental nor physical deficiency, and they were HIV negative with a good clinical status. Their violent or illegal behaviour were linked to the search for substances; therefore, in such a population, social and health problems were clearly prevalent than the criminal attitude. All inmates were involved in the standard rehabilitation program described above.

Recruitment of inmates for D.A.T. program was on a voluntary basis with the following limitations: they needed to be declared free of allergies towards dog fur, they should not be affected by dog phobia, and they must be free of religious or social prejudices against these animals and without pending accuse of animal abuse. In addition, they should have entered the institute since at least six months ago. The control group was composed without applying any matching, enrolling on voluntary basis inmates (of the cohort population considered) who had entered the institute since at least six months like the treated group and who were not interested in taking part to the D.A.T. program. The control group followed the standard rehabilitation program described above that did not include D.A.T. sessions.

### 2.6. Sample Size

Twenty-two inmates were selected among 35 people. The sample size was limited by the number of prisoners with eligibility criteria who agreed to take part in the study and by the small size of the D.A.T. program, which could involve a maximum of 12 inmates.

### 2.7. Data Collection

One week before the beginning (*T*_0_) and one week after the end (*T*_1_) of the D.A.T. program cases and controls were submitted to two psychological tests: Symptom checklist-90-revised (SCL-90-R) and Kennedy Axis V, used in the psychodiagnostic profiling activities provided by the rehabilitation program.

SCL-90-R is a 90-item self-report symptom inventory to measure psychological distress [34], which shows good internal consistency for all subscales and global scale in prison inmates [35]. Our analysis considered the ten primary distress symptom dimensions that include somatization, obsessive-compulsive symptoms, interpersonal sensitivity, depression, anxiety, hostility, phobic anxiety, paranoid ideation, psychoticism, sleep disorders and the global severity index.

Kennedy Axis V is composed of seven subscales that capture the clinician’s impression of the individual’s overall “Level of functioning” during the week before the treatment in the following areas: psychological impairment, social skills, violence, occupational skills, substance abuse, medical impairment and ancillary impairment [36]. In our study, the subscale “substance abuse” was replaced by “substance craving” to adapt the test at the prison context.

Collection of data and data entry were managed by the psychologist staff of the prison: they routinely evaluated all inmates guarded in the attenuated custody institute and systematically recorded data; therefore, SCL-90-R and Kennedy Axis V results were collected in a standardized way, according to their internal protocol.

### 2.8. Data Analysis

All data were entered into an Excel database and were analyzed using STATA 12.1 (StataCorp LP, College Station, TX, USA).

To evaluate the association between categorical variables (such as type of program and educational level), a chi-square test was used, or a Fisher's exact test if one of the expected cells is below 5.

To compare quantitative variables between two independent samples (D.A.T. vs. standard program), the non-parametric Wilcoxon rank-sum (Mann–Whitney) test was used on the differences *T*_0_-*T*_1_, SCL-90-R data not being normally distributed (Shapiro–Francia test’s *p*-value < 0.05), after having evaluated the omoschedasticity through a non-parametric Levene’s test (W50 > 0.05). For Kennedy data that followed, instead, the normal distribution, Student’s *t*-test for two independent samples was used, after having evaluated the omoschedasticity trough parametric Bartlett’s test (*p*-value > 0.05). Wilcoxon signed-ranks test or Student’s *t*-test for matched data were performed to evaluate differences between pre-post data (pre = one week before the beginning; post = one week after the end of D.A.T. sessions).

## 3. Results

### 3.1. Study Population

**Figure 2:**
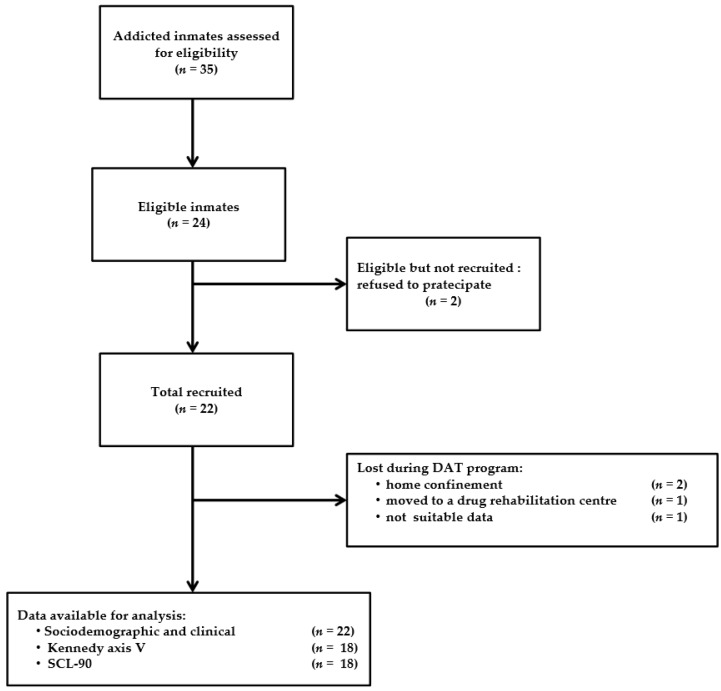
Participant flow diagram for the case-control study.

Among the 35 inmates evaluated, only 24 satisfied eligibility criteria. Twenty-two of them were accepted to take part in the study. Twelve inmates were enrolled for D.A.T. sessions and 10 composed the control group. Not all inmates enrolled in the treated group completed the D.A.T. program: three of them left the attenuated custody institute before the end of the study; two obtained the home confinement and one was moved in a drug rehabilitation centre. Moreover, one of the inmates enrolled in the control group was excluded from statistical analysis because data collected resulted in being an outlier for most parameters considered (Figure 2). The demographic characteristics of patients enrolled are summarized in Table 1. All of them had a history of substance abuse longer than 36 months and their median prison stay was more than 12 months at the beginning of the program. Nine (77.7%) cases and six (55.5%) controls followed a pharmacological treatment with benzodiazepines, phenothiazine and antidepressants. There are no statistical differences in age, educational level and prison stay between treated and control inmates; therefore, the two groups are comparable (*p* > 0.05) (Wilcoxon’s *p*-value = 0.4018; Fisher’ *p*-value = 1.00; Chi-squared test’s *p*-value = 0.133, respectively).

### 3.2. Test Analysis

SCL-90-R data analysis highlighted a significant reduction in symptoms between *T_0_* and *T_1_* in the treated group (Student’s *t*-test for matched data: *p*-value < 0.05) except for three subscales: phobic anxiety (*p*-value = 0.07), hostility (*p*-value = 0.42) and interpersonal sensitivity (*p*-value = 0.17) (Table 2). In the control group, data didn’t have a normal distribution; therefore, we applied the Wilcoxon signed-rank test achieving a significant reduction in symptoms from *T*_0_ to *T*_1_ only for sleep disorders (*p*-value = 0.04) (Table 3).

**Figure 3:**
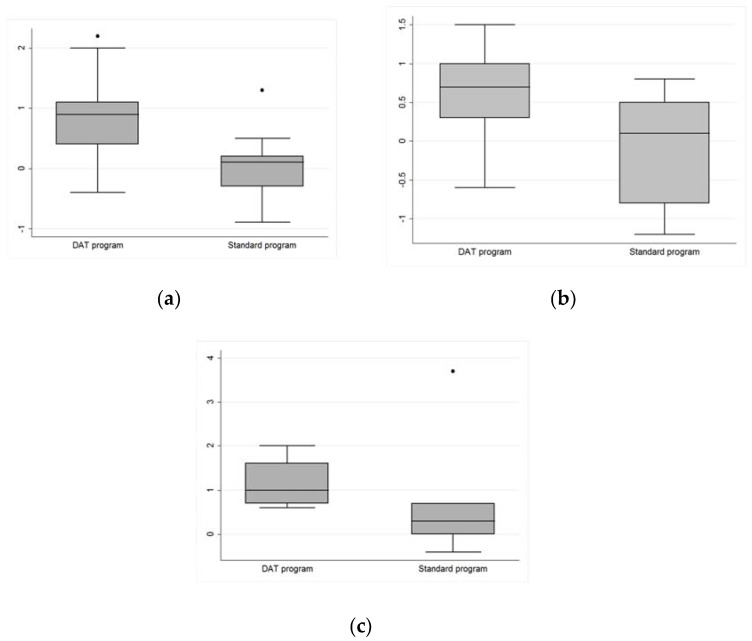
(**a**) SCL-90-R. Significant reduction of depression symptoms comparing inmates involved in the D.A.T. program and the control group (*p*-value < 0.05); • = outlier values; (**b**) SCL-90R. Significant reduction of paranoid ideation symptoms comparing inmates involved in the D.A.T. program and the control group (*p*-value < 0.05); (**c**) SCL-90R. Significant reduction of sleep disorders comparing inmates involved in the D.A.T. program and the control group (*p*-value < 0.05); • = outlier value.

**Figure 4:**
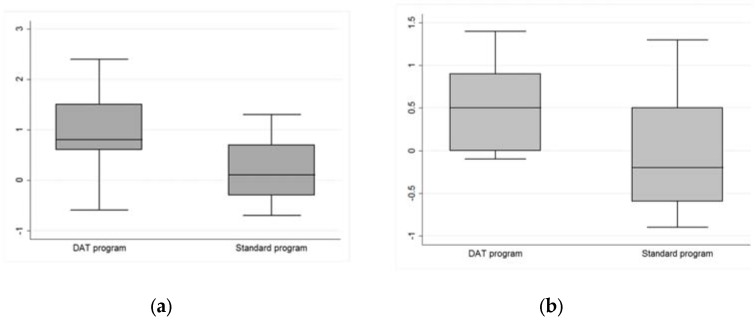
(**a**) SCL-90-R. Significant reduction of anxiety symptoms comparing inmates involved in the D.A.T. program and the control group (*p*-value < 0.1); (**b**) SCL-90R. Significant reduction of psychotic symptoms comparing inmates involved in the D.A.T. program and the control group (*p*-value < 0.1).

Moreover, the Wilcoxon rank-sum (Mann-Whitney) test for two independent samples pointed out a reduction of symptoms significantly different between treated and control group for the global severity index (*p*-value = 0.063). In particular, as reported in Figure 3 and Figure 4, five subscales showed median values higher in the treated group: depression, paranoid ideation and sleep disorders with *p*-value < 0.05, anxiety and psychoticism with *p*-value < 0.10 (Table 4). If we consider Bonferroni correction, however (adjusted alfa = 0.05/10 items), none of the subscales shows significant differences between D.A.T. and standard rehabilitation program.

Kennedy Axis V data analysis showed an improvement comparing *T*_0_ and *T*_1_ in both cases and controls for all areas considered by the test, except for medical impairment in the D.A.T. group (Wilcoxon signed-rank test’s *p*-value = 0.6376) (Table 5, Table 6).

Collected data followed a normal distribution so we applied a Student’s *t*-test for two independent samples, finding that the enhancement of patients involved in D.A.T. was significantly higher than in the control group for psychological impairment and social skills (*p*-value < 0.05) (Table 7).

## 4. Discussion

The purpose of this study was to explore the potential effect and efficacy of a D.A.T. program as co-therapy to enhance the standard rehabilitation program directed to detained drug users of the attenuated custody institute of “Due Palazzi” prison in Padua (Italy). Our findings suggest that D.A.T. strengthens the therapeutic impact of the standard rehabilitation program on mood and behaviour of inmates, improving their psychological functioning and reducing some dysfunctional symptoms.

### 4.1. Functioning and Social Skills

Strong associations between substance abuse and social alienation and personality disorders concerning social behaviour are documented by several studies [37,38,39,40]. Considering addicted inmates, a diagnosis of antisocial personality disorders is about 10 times more likely than in the general population [41]. Moreover, some studies showed that these patients have a higher tendency to perceive the external world as hostile and to consider others as responsible for their own problems, showing a psychological profile characterized by paranoid ideation and an avoidant defensive style that easily results in antisocial behaviour and/or deviant behaviour [42,43]. Our results corroborate with those found in the literature both from the more general companion animal ownership research [42,43,44,45] and from other specific studies about prisoners [20,24,46,47]. Indeed, the effects on patients’ psychological functioning in social situations highlighted by therapists through Kennedy Axis V were sustained by the significant reduction of symptoms linked to the SCL-90-R dimensions of paranoid ideation and psychoticism in inmates involved in D.A.T. compared to those who followed only the standard rehabilitation program. Therefore, our findings highlighted again the potentiality of D.A.T. addressed to this target population in enhancing social skills acting on psychological dimensions of major interest to improve their psychopathological profile. Moreover, we pointed out that inmates improved their ability in dealing with daily social situations. Anyway, we didn’t notice a reduction in psychological symptoms linked to interpersonal sensitivity and hostility reported by the SCL-90-R. Their feelings of anger and rage against the context in which they lived as well as their discomfort during interactions with people were not mitigated, probably because of their antisocial traits.

### 4.2. Craving

Much research in both human and animal models of addiction highlighted that the transition from controlled to compulsive drug seeking and taking is strongly conditioned by craving [5,8,48,49]. It can lead to heavier use and abuse of substances through an inability to regulate consumption or may induce a change in use patterns [50]. Furthermore, available evidence demonstrates an association between chronic consumption of addictive substances and heightened craving [51,52,53], which is also confirmed in an incarcerated population [54]. Moreover, some studies have shown that craving is predictive of future relapse in substance use or misuse [7,55,56]. Therefore, the cognitive therapy for substance abuse includes training to improve craving control and emotion management [57]. In this framework, the D.A.T. program showed its potentiality to reduce craving in this patient category. Kennedy Axis V results pointed out a significant reduction in violence and craving of D.A.T. involved inmates, and these data were confirmed by a reduction in symptoms linked to SCL-90-R obsessive and compulsive behaviour dimension even if the reduction was not significant compared to the control group, maybe because of the small sample number. This evidence further supports the perspective to introduce D.A.T. as co-therapy in rehabilitation programs addressed to drug addicted inmates.

### 4.3. Psychological Dysfunction Symptoms

The positive impact of animal assisted therapy on outcomes like major depression and anxiety symptoms were frequently highlighted on different kinds of patients: children [58,59], psychiatric inpatients [60,61,62,63], hospitalized women with at risk pregnancy [64], and elderly institutionalized patients [65,66,67]. The few research works conducted in the prison setting reported an improvement in inmates’ moods thanks to D.A.T. and a decrease in salivary cortisol values after D.A.T sessions [29,46].

Our findings, deriving from the analysis of Kennedy Axis V results, confirmed a positive decrease in anxiety as well as the depression of inmates involved in the D.A.T. program. Differences were significant compared to the control group and were confirmed by SCL-90-R results. They also pointed out a strong reduction in sleep disorders, symptoms considered strictly related to depression and anxiety. These results are particularly interesting considering that they were reached despite the prison context. These positive findings are probably due to two D.A.T. mechanisms. Indeed, the human-animal relationship improves both the perceived social support [68,69,70,71,72,73,74] and the learning of active coping strategies [75,76], which are protective factors against psychological distress whose well-known related outcomes are anxiety and depression [77,78,79,80]. Moreover, the SCL-90-R Global Severity Index confirmed a significant positive change in the psychological distress level of inmates, which could take advantage from the D.A.T. sessions.

### 4.4. Limitations of the Study

Randomized control trials (R.C.T.) are the gold standard to determine the effect of an animal-assisted intervention on a certain population and a certain outcome [81,82], but it is not always possible to build an effective R.C.T., especially in psychotherapy or behavioural research [83] and when you have to operate in a difficult setting such as prison. Therefore, we try to build a pilot study with a controlled design exploiting the opportunity provided by the health care service of the attenuated custody institute of the Italian prison “Due Palazzi” that decided to add D.A.T. sessions to its standard rehabilitation program addressed to male addicted inmates. The study design aimed to answer the practical question of whether D.A.T. may be added-on permanently to what clinics currently do in this facility. Our study design had to take into account both practical and ethical issues: all limitations due to the prison setting, the high turn-over of the inmates housed in the institute and their small number as well as the limited financial support. Only 22 inmates accepted to enter the study and there were not the terms to build a novelty group. D.A.T. and control group were built on voluntary basis.

The limited financial support ensured to have a D.A.T. program of six months with possible extension year by year according to financial means. Therefore, D.A.T. was included in the rehabilitation program as an optional co-therapy. It is quite well known that psychological treatments are most successful when patients can choose them because they are more motivated to comply with treatments they prefer [84] and this is the case. However we try to balance this bias building a control group that was not interested in D.A.T. and chose to be involved in the standard treatment. Therefore, the treated and control groups were made up with inmates who were equally well motivated to be engaged.

We are aware that our results can’t be generalized and there is a lack of external validity; indeed, our statistical power is only 0.25 for a SCL-90-R test but almost 0.80 for the Kennedy axis V; therefore, some non-significant results could become statistically significant testing a bigger number of inmates. Anyway, we think that these data have ecological validity considering the study design and the methods applied.

Finally, we consider the absence of follow up on another critical point of our study, but most of the inmates involved left the institute in a short time after the end of D.A.T. sessions, making the follow up unachievable; therefore, we can’t determine if changes in outcomes led to long-term benefits for patients involved.

## 5. Conclusions

This pilot study results extend previous findings on D.A.T. effects on a specific population, drug addicted male inmates, showing the useful and significant support of D.A.T. in enhancing their social skills, reducing distress levels and, as a consequence, depression and anxiety symptoms in a highly stressing environment like prison. Despite the small sample size, the absence of randomization and the lack of follow up, the encouraging results that we obtained suggest to us to keep the mainstreaming of our standard rehabilitation program with a structured D.A.T. program in the perspective to collect additional data to support our preliminary findings. We conclude that larger and multicentre studies with follow-up assessment are needed to more definitively explore the beneficial and long-lasting effects of D.A.T. on this target population.

## Figures and Tables

**Table 1:** Sociodemographic characteristics and drug use of participants to the study.

Demographic Characteristics	Dog Assisted Therapy (D.A.T.) Program (*n* = 12)	Standard Program (*n* = 10)
Age (mean ± SD in years)	35.5 ± 13.83	42.9 ± 9.1
Primary School Education (%)	22.2	0
Secondary School Education (%)	77.8	88.9
High School Education (%)	0	11.1
Degree or PhD (%)	0	0
Prison Stay (median in months)	12	12
Pharmacological treatment (%)	77.7	55.5

**Table 2:** D.A.T. group. SCL-90-R results.

Variable	Mean ± SD		*t*-Student
	*T*_0_	*T*_1_	*p*-Value
Somatization	0.98 ± 0.89	0.21 ± 0.24	0.0227
Obsessive-compulsive symptoms	1.07 ± 0.61	0.46 ± 0.29	0.0102
Interpersonal sensitivity	0.60 ± 0.59	0.23 ± 0.24	0.1754 *
Depression	1.34 ± 0.84	0.45 ± 0.32	0.0139
Anxiety	1.39 ± 0.95	0.44 ± 0.35	0.0130
Hostility	0.57 ± 0.58	0.43 ± 0.36	0.4153 *
Phobic anxiety	0.46 ± 0.55	0.06 ± 0.07	0.0703 *
Paranoid ideation	1.17 ± 0.72	0.54 ± 0.49	0.0175
Psychoticism	0.73 ± 0.62	0.19 ± 0.16	0.0173
Sleep disorders	1.78 ± 0.53	0.63 ± 0.59	0.0002
Global severity index	1.01 ± 0.54	0.35 ± 0.19	0.0056

* *p*-value > 0.05.

**Table 3:** Control group. SCL-90-R results.

Variable	Mean ± SD		Wilcoxon Signed-Rank Test
	*T*_0_	*T*_1_	*p*-Value
Somatization	1.17 ± 1.30	0.65 ± 0.74	0.2207
Obsessive-compulsive symptoms	1.37 ± 1.05	0.83 ± 0.53	0.2026
Interpersonal sensitivity	0.70 ± 0.51	0.52 ± 0.55	0.5403
Depression	1.10 ± 0.77	0.83 ± 0.48	0.6101
Anxiety	1.07 ± 0.83	0.73 ± 0.41	0.3580
Hostility	0.67 ± 0.75	0.53 ± 0.54	0.7208
Phobic anxiety	0.82 ± 1.40	0.35 ± 0.51	0.2578
Paranoid ideation	0.86 ± 0.74	0.83 ± 0.59	0.9188
Psychoticism	0.84 ± 0.77	0.66 ± 0.41	0.6831
Sleep disorders	1.89 ± 1.47	1.00 ± 1.02	0.0411 *
Global severity index	1.00 ± 0.82	0.67 ± 0.43	0.5400

* *p-*value < 0.05.

**Table 4:** Comparison between D.A.T. and Control group (Δ*T_1_*-*T_0_*). SCL-90-R results.

Variable	Mean ± SD		Wilcoxon Signed-Rank Test
	D.A.T. Group	Control Group	*p*-Value
Somatization	0.78 ± 0.78	0.29 ± 0.92	0.2152
Obsessive-compulsive symptoms	0.61 ± 0.55	0.26 ± 0.77	0.2881
Interpersonal sensitivity	0.36 ± 0.71	0.07 ± 0.75	0.5346
Depression	0.89 ± 0.84	0.02 ± 0.65	0.0468 *
Anxiety	0.94 ± 0.89	0.16 ± 0.73	0.0562 **
Hostility	0.17 ± 0.50	−0.04 ± 0.66	0.4772
Phobic anxiety	0.37 ± 0.56	0.32 ± 1.31	0.1133
Paranoid ideation	0.64 ± 0.62	−0.13 ± 0.76	0.0272 *
Psychoticism	0.54 ± 0.55	0.02 ± 0.78	0.0926 **
Sleep disorders	1.13 ± 0.51	0.62 ± 1.22	0.0201 *
Global severity index	0.76 ± 0.45	0.12 ± 0.67	0.0627 **

* *p-*value < 0.05; ** *p-*value < 0.1.

**Table 5:** D.A.T. group. Kennedy Axis V results.

Variable	Mean ± SD		Wilcoxon Signed-Rank Test
	*T*_0_	*T*_1_	*p*-Value
Psychological impairment	60.42 ± 7.22	69.17 ± 8.48	0.0017
Social skills	58.75 ± 10.25	68.33 ± 12.85	0.0018
Violence	62.92 ± 10.10	70.42 ± 10.97	0.0021
Occupational skills	64.17 ± 9.96	70.83 ± 12.94	0.0027
Substance craving	59.17 ± 13.29	67.50 ± 13.57	0.0042
Medical impairment	83.33 ± 10.73	82.92 ± 9.16	0.6376 *
Ancillary impairment	60.00 ± 8.53	65.83 ± 8.75	0.0026

* *p-*value > 0.05.

**Table 6:** Control group. Kennedy Axis V results.

Variable	Mean ± SD		Wilcoxon Signed-Rank Test
	*T*_0_	*T*_1_	*p*-Value
Psychological impairment	63.75 ± 8.82	69.17 ± 10.62	0.0058
Social skills	64.17 ± 9.00	68.33 ± 11.15	0.0115
Violence	72.50 ± 14.22	76.25 ± 12.64	0.0261
Occupational skills	65.83 ± 13.11	69.58 ± 14.37	0.0052
Substance craving	62.50 ± 9.65	66.67 ± 10.08	0.0056
Medical impairment	72.08 ± 11.57	77.08 ± 12.69	0.0092
Ancillary impairment	62.08 ± 8.65	66.67 ± 10.73	0.0111

**Table 7:** Comparison between D.A.T. and Control group (Δ*T*_1_-*T*_0_). Kennedy Axis V results.

Variable	Mean ± SD		*t*-Student
	D.A.T. Group	Control Group	*p*-Value
Psychological impairment	8.75 ± 3.11	5.42 ± 4.50	0.0464 *
Social skills	9.58 ± 4.50	4.17 ± 4.17	0.0058 *
Violence	7.50 ± 5.00	3.75 ± 4.83	0.0750
Occupational skills	6.67 ± 5.36	3.75 ± 3.11	0.1174
Substance craving	2.08 ± 5.82	4.17 ± 3.59	0.3028
Medical impairment	−0.42 ± 6.89	5.00 ± 4.77	0.0356
Ancillary impairment	5.83 ± 3.59	4.58 ± 4.50	0.4599

* *p-*value < 0.05.
